# Direct Observation of a Roaming Intermediate and Its Dynamics

**DOI:** 10.1021/jacs.4c01543

**Published:** 2024-04-29

**Authors:** Grite L. Abma, Michael A. Parkes, Weronika O. Razmus, Yu Zhang, Adam S. Wyatt, Emma Springate, Richard T. Chapman, Daniel A. Horke, Russell S. Minns

**Affiliations:** † Institute for Molecules and Materials, Radboud University, Heijendaalseweg 135, Nijmegen 6525 AJ, The Netherlands; ‡ Department of Chemistry, University College London, 20 Gordon Street, London WC1H 0AJ, U.K.; § School of Chemistry, University of Southampton, Highfield, Southampton SO17 1BJ, U.K.; ∥ Central Laser Facility, STFC Rutherford Appleton Laboratory, Didcot, Oxfordshire OX11 0QX, U.K.

## Abstract

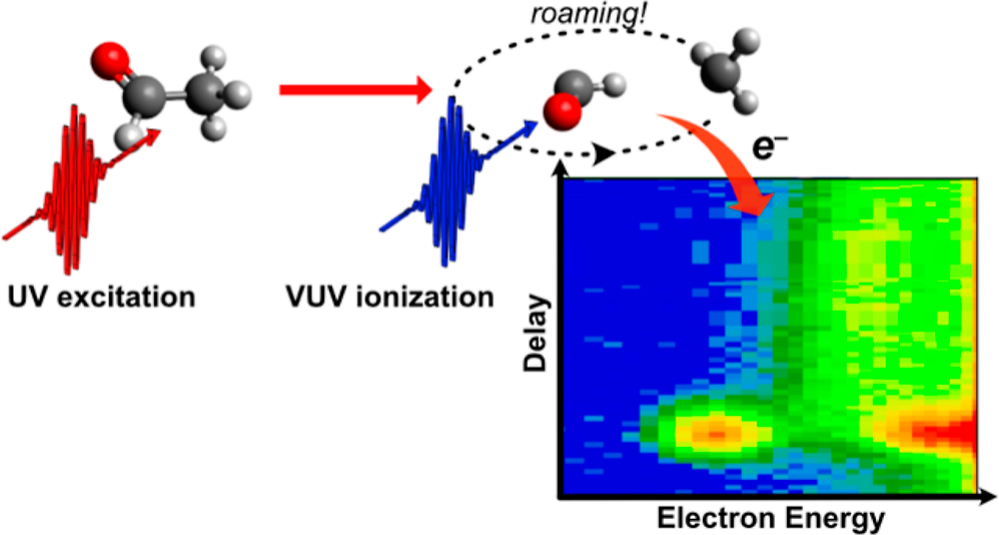

Chemical reactions are often characterized by their transition state, which defines the critical geometry the molecule must pass through to move from reactants to products. Roaming provides an alternative picture, where in a dissociation reaction, the bond breaking is frustrated and a loosely bound intermediate is formed. Following bond breaking, the two partners are seen to roam around each other at distances of several Ångstroms, forming a loosely bound, and structurally ill-defined, intermediate that can subsequently lead to reactive or unreactive collisions. Here, we present a direct and time-resolved experimental measurement of roaming. By measuring the photoelectron spectrum of UV-excited acetaldehyde with a femtosecond extreme ultraviolet pulse, we captured spectral signatures of all of the key reactive structures, including that of the roaming intermediate. This provided a direct experimental measurement of the roaming process and allowed us to identify the time scales by which the roaming intermediate is formed and removed and the electronic potential surfaces upon which roaming proceeds.

## 1 Introduction

The traditional view of a chemical reaction relies on transition state theory, where specific structures act as bottlenecks through which reactants must pass to form products. Over the course of the past 15 years, a new type of photodissociation reaction mechanism, termed “roaming”, has offered an alternative to this view and is thought to compete with conventional transition state-driven dynamics.^1−6^ This roaming pathway can occur when, en route to dissociation, a shallow plateau in the potential energy surface is encountered, leading to the formation of a quasi-bound complex of two fragments.

Roaming was first discovered in formaldehyde, in which an intermediate is formed by a hydrogen atom roaming around a HCO radical.^1^ Since this first observation, the roaming pathway has been experimentally shown in several systems and is thought to be nearly ubiquitous in photodissociation reactions.^4,7,8^ Direct experimental observation of the roaming intermediates is however challenging, meaning that roaming processes have largely been identified through the detailed analysis of the energy partitioning detected in the final reaction products, and correlations in the usually bimodal internal energy distribution of the fragments have been used as evidence of roaming processes.^2,3,5,9^

The challenge to experiment is that the characteristics of roaming intermediates place extreme constraints on the measurement processes used. By its very nature, the occurrence of roaming produces a molecular complex that has an impossible to define geometry as the reaction intermediate. The roaming partners can take on a wide range of structures defined by a broad plateau on the potential energy surface. The typically rapid formation of the intermediate and subsequent slow decay necessitates measurements on time scales covering many orders of magnitude, potentially from femtoseconds to nanoseconds. These characteristics provide a demanding backdrop to experiment and theory alike. Accurate theoretical descriptions of the roaming wavepacket are difficult to perform, while the identification of specific structural or spectral probes is challenging to experiments.

Experimental probes that are sensitive to all aspects of the roaming dynamics are needed, ideally capturing the formation and decay of the roaming intermediate as well as the final products formed. To the best of our knowledge, the first experimental observation of a roaming intermediate has only recently been reported. Utilizing a time-resolved Coulomb explosion imaging measurement with coincident detection of multiple fragment ions, the authors were able to identify a signature of the first step in the roaming process in formaldehyde, as well as the formation of the intermediate complex on a time scale of a few hundred femtoseconds.^6^ However, a full time-resolved measurement of roaming dynamics and of the eventual fate of the intermediate has so far not been reported.

Herein, we report on the direct and time-resolved observation of the formation and subsequent decay of the roaming intermediate in the photodissociation of acetaldehyde (CH_3_CHO). Here, UV absorption leads to carbon–carbon bond breaking and the formation of CH_3_ and HCO radicals. The radicals begin to separate and can either follow a pathway that is well described by conventional transition state theory and rapidly leads to the separation of the two radical fragments or a roaming pathway where the two fragments are seen to remain in relative close proximity (at C–C distances of 3–4 Å^4^) for an extended period of time. For the molecules that follow the roaming pathway, the two radicals explore a wide range of configurations before reaching one that allows for the reaction of the two fragments, forming CH_4_ and CO, or dissociation, leading to the relatively delayed formation of the radical products. The roaming channel in this photodissociation has been studied by various groups both theoretically^10−12^ and experimentally^3,9,13−19^ and at a vast range of dissociation energies from ∼220 to 330 nm.^17^ The primary experimental observable in all these studies was the energy distribution of final radical (HCO, CH_3_) or molecular (CO, CH_4_) products, meaning a direct observation of the roaming intermediate, and its formation pathway, has so far remained difficult to measure. Existing time-resolved studies lack the necessary temporal resolution to observe the crucial first electronic dynamics following photoexcitation.^14,15^ Here, we study the ultrafast dynamics following excitation of acetaldehyde at 262 nm (4.7 eV), populating the first electronically excited state (S_1_) via a π* ← *n* transition.^20^ At this excitation energy, the dissociation mechanism is not well understood, with some studies invoking intersystem crossing to lower-lying triplet states as a potential relaxation pathway,^14,19^ while others suggest that at these pump wavelengths, the available energy is sufficient to overcome a barrier on the S_1_ surface, leading to internal conversion via a conical intersection with the singlet ground state S_0_.^10,11,16−18^ Following internal conversion, the system can either directly dissociate, or it can lead to roaming by exploring a wide plateau on the potential surface, eventually leading to both radical and molecular dissociation channels. To directly observe the formation and subsequent destruction of the roaming intermediate, we present here a photoelectron spectroscopy experiment which utilizes a femtosecond extreme ultraviolet (XUV) probe pulse.^21^ The sensitivity of photoelectron spectroscopy to changes in the bonding character, combined with the high energy of the probe, allowed us to monitor all aspects of a roaming reaction.^22,23^ The measurements captured spectroscopic signals that are only consistent with the formation and removal of roaming intermediates as well as the formation of the HCO radical product via direct and roaming reaction pathways. The experimental data is supported by ab initio calculations of the potential energy surfaces and simulated photoelectron spectra, which allows us to identify the electronic state upon which roaming proceeds.

## 2 Results and Discussion

**Figure 1:**
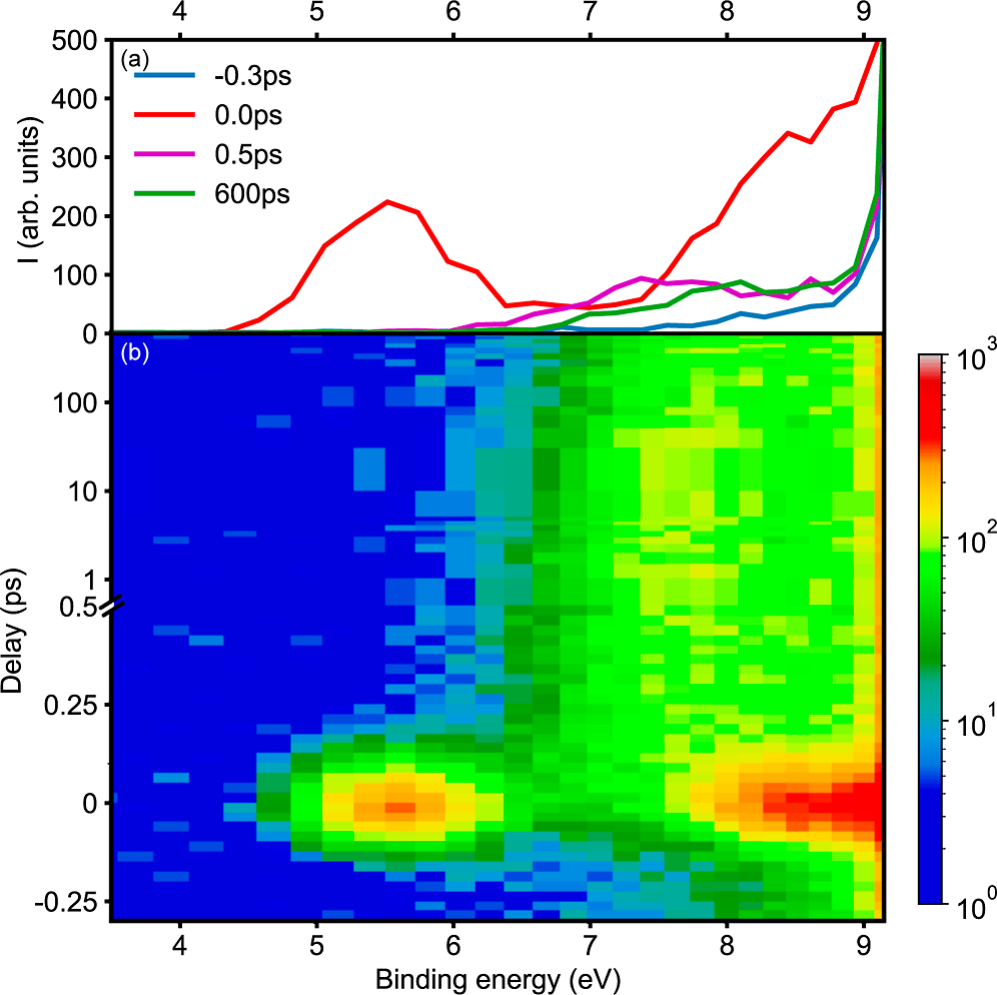
Time-resolved photoelectron spectra of acetaldehyde following excitation at 262 nm and ionization at 56 nm (22.3 eV). (a) Selected spectra at different pump–probe delays, before *t*_0_ (blue), at *t*_0_ (red), at 500 fs (purple), and at long delays of 600 ps (green). (b) Surface plot showing the time evolution of the photoelectron spectra; note the mixed linear-logarithmic time delay (*y*) axis, which is linear up to 0.5 ps, and logarithmic thereafter.

Resulting photoelectron spectra are shown in Figure 1. Figure 1a shows characteristic photoelectron spectra at selected pump–probe delays. With pump and probe pulses temporally overlapped (*t*_0_, red trace), we observe two broad peaks centered around binding energies of 5.5 and 8.5 eV. These correspond to ionization of the initially excited state of the bound CH_3_CHO molecule into the ground (D_0_) and first electronically excited (D_1_) state of the ion, respectively. These features are very short-lived, and by 500 fs (purple trace), we observe only a very broad feature from around 6.0 eV to at least 9.0 eV binding energy; beyond this energy, any signals are obscured by the direct ionization of ground-state acetaldehyde, as further discussed in the Supporting Information. Going toward even longer delays (600 ps, green trace), this broad feature has narrowed and shifted toward higher binding energies and now corresponds to final reaction products, in this case, the formation of the HCO radical, as further discussed below.

In Figure 1b, we show the full time-resolved photoelectron spectrum and note the mixed linear-logarithmic scale on the time delay (*y*) axis. The full spectrum highlights the very short-lived nature of the initially excited state and the formation of a broad peak within a few hundred femtoseconds. On longer time scales of hundreds of picoseconds, this peak is seen to decrease in intensity on the low binding energy side.

**Figure 2:**
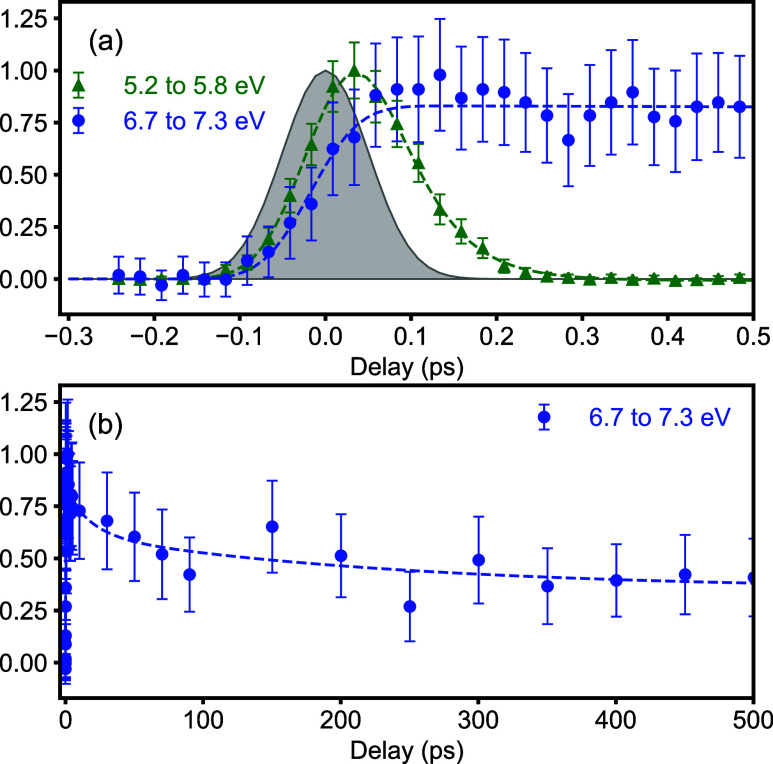
Time evolution of the observed photoelectron signal in selected energy regions on short (a) and long (b) time scales. Gray shading in (a) corresponds to the instrument response function, as discussed in the experimental details section of the Supporting Information.

To extract dynamic information, we integrate the time-resolved spectrum over selected binding energy slices, Figure 2. The green trace in Figure 2a shows the dynamics in the 5.2 to 5.8 eV binding energy region, corresponding to the initially excited state in acetaldehyde. A rapid exponential decay with a time constant of around 50 fs is observed, which we assign to the rapid depopulation of the initially excited S_1_ state. The blue traces in Figure 2a,b show the binding energy region of 6.7–7.3 eV. Following a fast rise, a slow decay on a time scale of a few hundred picoseconds is observed, until a plateau of around half the intensity is reached. This behavior clearly indicates the existence of multiple overlapping contributions to the photoelectron spectrum, belonging to a transient state decaying on the 100s of picosecond time scale, and a (much) longer-lived contribution likely from a reaction product. In order to deconvolve these, we perform a global 2D fit to the entire time-resolved photoelectron spectrum to extract so-called decay-associated spectra (DAS), which represent the spectral contribution to each underlying dynamical process.^24−26^ We require a total of 3 separate processes, each with its own associated lifetime, to accurately represent the data.

**Figure 3:**
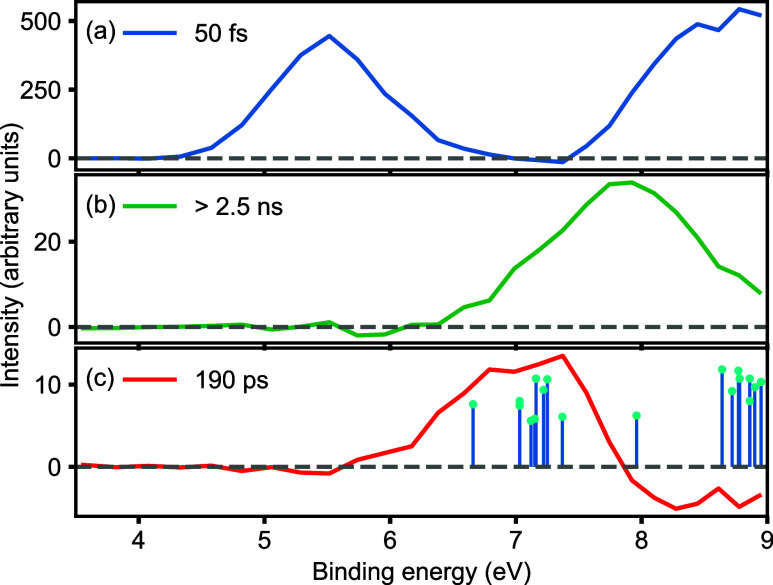
Decay-associated spectra extracted from the experimental data in Figure 1. Three time constants of 50 fs (a), >2.5 ns (b), and 190 ps (c) are required to accurately represent the data. In (c), calculated binding energies following ionization of the roaming intermediate in various geometries on the *S*_0_ state are indicated as sticks, with the length corresponding to the relative cross-section.

The resultant DAS are plotted in Figure 3 and have associated time constants of 50 fs, >2.5 ns, and 190 ps in Figure 3a–c, respectively. At early times, the dynamics are dominated by the spectral contribution shown in the top panel of Figure 3. The broad peaks correlate well with the expected binding energies for ionization from the Franck–Condon geometry of the initially excited S_1_ state. These peaks have an associated time constant of approximately 50 fs, meaning this is an extremely short-lived configuration of the molecule.

In Figure 3b, we show the spectral component that has an associated lifetime of >2.5 ns. This is much longer than the time scale of our experiment and hence can be considered an “infinite lifetime”, such that on the time scale measured, the intensity remains unchanged, and this corresponds to one of the products of the reaction. Based on the known reaction products and their ionization energies (CH_3_ 9.84 eV, HCO 8.1 eV, CH_4_ 12.61 eV, CO 14.01 eV),^27^ we assign this feature to the rapid formation of HCO, occurring within the first 100 fs after excitation. As the HCO fragment can only be formed on the electronic ground state, the appearance of this product also indicates that the internal conversion from S_1_ to S_0_ occurs on this ultrafast time scale.

The lower panel in Figure 3 shows a more complex spectrum, including regions of positive and negative amplitudes, with an associated time constant of 190 ± 10 ps. The uncertainty corresponds to a statistical 2σ standard error extracted from the DAS fitting; further details are given in the Supporting Information. In DAS, regions of positive amplitude relate to spectral features that decay on the associated time scale (as seen in Figure 3a,b), while negative amplitudes relate to spectral features that increase in intensity on that time scale. The observation of both positive and negative amplitudes within the DAS is therefore a direct sign for the flow of population from one region of the spectrum, where positive amplitude is observed, to regions of the spectrum with negative amplitude. Figure 3c therefore clearly shows the decay of a species with binding energies in the range 5.3–7.6 eV into a new species with higher binding energies on a time scale of 190 ps. At these higher binding energies, the signal strongly overlaps with the observed HCO peak (Figure 3b), indicating that we are producing additional HCO fragments over this much longer time scale, in addition to those produced from the rapid initial dissociation. The observed feature in the 5.3–7.6 eV range therefore corresponds to a reactive intermediate, formed within the first few hundred femtoseconds after excitation and decaying on a 190 ps time scale to yield (as one product) the HCO radical. We therefore assign this transient species to the roaming intermediate CH_3_···HCO. The observed binding energy is indicative of an intermediate structure between bound CH_3_CHO and the radical product HCO, suggesting that we are monitoring a range of structures associated with the weakly bound roaming intermediate. The observed rapid appearance of the intermediate within the first few hundred femtoseconds agrees well with recent observations in formaldehyde.^6^

Previous measurements have suggested the involvement of intersystem crossing (ISC) to the triplet states,^18,28−30^ which could also explain the observation of a photoelectron peak at these intermediate energies. We find this unlikely in the present situation due to the temporal characteristics of the observed spectral changes. For a molecule like acetaldehyde, which contains no heavy atoms, such rapid (sub 100 fs) and efficient ISC into the triplet manifold is extremely unlikely. The spin–orbit (SO) coupling matrix element that facilitates ISC between S_1_ and S_0_ is approximately 10 cm^–1^.^31^ This means that direct transfer from S_1_ to T_1_ on the measured lifetime of the S_1_ state can be ruled out. Subsequent transfer from S_0_ to T_1_ is theoretically possible and has a larger associated SO-coupling of approximately 70 cm^–1^. This process is energetically less favorable and would involve transfer onto a state with higher potential energy. The fact that the photoelectron band is formed extremely rapidly but subsequently remains unchanged indicates that there are no electronic structure changes on the time scale of the measurement. We therefore suggest that at the 262 nm pump wavelength used here, we excite into the S_1_ state above the barrier to the S_1_/S_0_ conical intersection.^10,11,16−18^ Excitation above the barrier leads to rapid internal conversion to S_0_ and the subsequent role of the triplet states is therefore negligibly small. We note that this assignment agrees with that suggested in previous experimental and theoretical studies.^17,32^ At longer wavelengths, where the barrier on S_1_ cannot be overcome, transitions to T_1_ will become important and play a significant role in the reaction, as shown by a large number of studies performed at wavelengths longer than 300 nm.^9,18,33^

**Figure 4:**
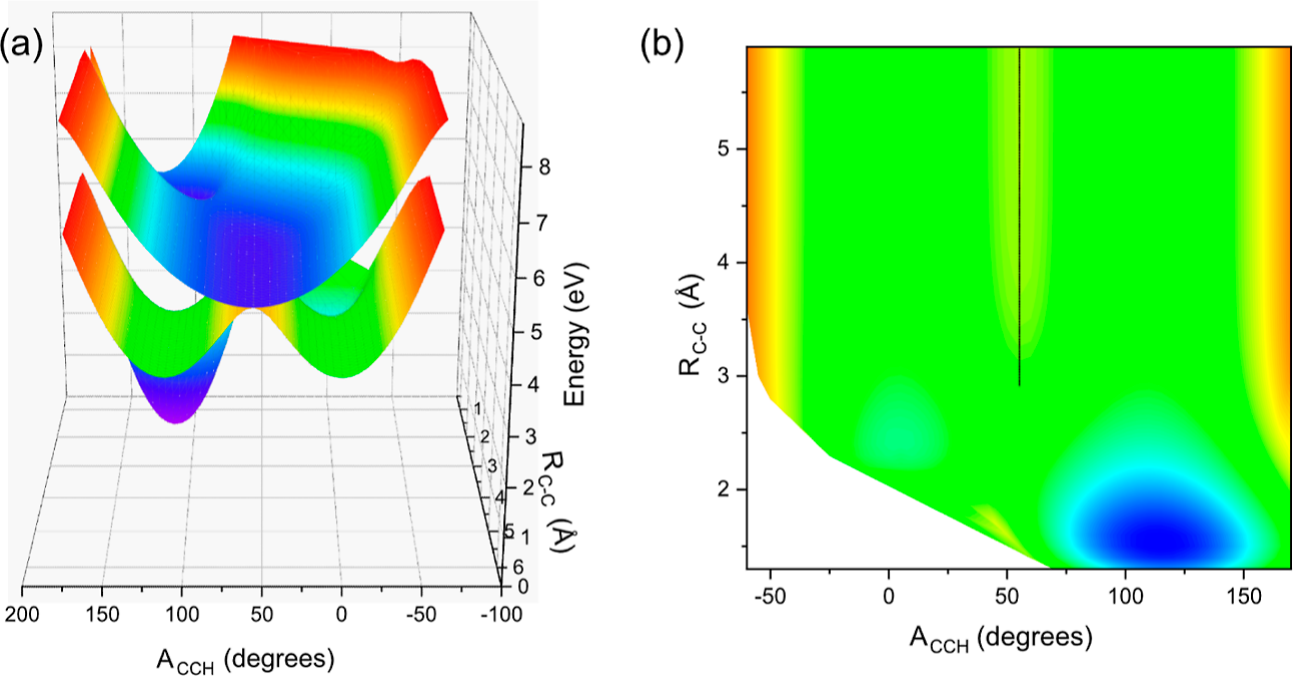
Calculated potential energy surfaces of acetaldehyde. (a) Projection of the S_1_ and S_0_ surfaces along the C–C bond length and CCH bond angle coordinates. (b) Contour plot of the S_0_ ground-state surface; the dashed line indicates a conical intersection seam with the S_1_ state.

In order to support our assignments, and to understand where on the potential energy surface roaming takes place, we calculated the coupled potential energy surfaces important to the dynamics; technical details are given in the Supporting Information. The acetaldehyde potential energy surface was explored at a range of C–C bond lengths and C–C–H bond angles. A projection of the initially excited S_1_ and ground-state S_0_ surfaces is plotted in Figure 4a, while Figure 4b shows a contour projection of the S_1_ surface. The figure shows an extended crossing (points of degeneracy) between the two surfaces at a C–C–H angle of 50° with C–C bond lengths of greater than 2.5 Å (indicated by the dashed line in Figure 4b). Efficient population transfer is facilitated at these crossing points (conical intersection seam) allowing for rapid repopulation of the electronic ground state. Surrounding this crossing associated with the conical intersection is a broad and flat plateau extending over a wide range of angles and bond lengths. We note that within the figure, we can only provide a limited projection of the full potential energy surface (which would contain 15 dimensions), and this plateau extends over a much wider range of geometries than can be plotted within a single projection.

We have performed full quantum trajectory simulations covering the first 100 fs to confirm that ultrafast internal conversion is viable; see Supporting Information for details. The calculated electronic state populations show a rapid transfer from S_1_ to S_0_ occurring within 20 fs. This is in line with the 50 fs time constant observed experimentally for the decay of the initially excited state and confirms our assignment of ultrafast internal conversion as the primary step in the dynamics.

While we cannot perform trajectory simulations for the full-time frame of the experiment, the calculated ground- and excited-state potential energy surfaces provide us with a landscape upon which we can obtain theoretical photoelectron spectra for a variety of geometries associated with the initially excited Franck–Condon geometry, the dissociated fragments, and the roaming intermediate. The geometries chosen for calculation of the roaming signal were based on the positions of the conventional and roaming transition state as reported by Bowman.^34^ Fixed and relaxed geometry potential energy scans were then performed around these starting points, and representative structures from these scans were taken forward for calculation of their photoelectron spectrum. Full details on the calculations can be found in the Supporting Information as we limit the presentation here to the calculated photoelectron energies for ionization of the roaming intermediate on the ground electronic state. Calculated energies and relative cross sections for various geometries in the broad plateau region are shown in Figure 3c, overlaid with the experimental photoelectron spectrum assigned to the roaming intermediate. The calculations show that a broad range of binding energies are possible, correlating with the “floppy” structure of the molecule and the large regions of the potential energy surface explored. Importantly, several calculated photoelectron energies agree remarkably well with the feature assigned to the roaming intermediate, in the range of ∼6–8 eV binding energy, giving us confidence in the assignment of this feature to the roaming intermediate. A further cluster of expected photoelectron peaks is toward higher binding energies of >8 eV. Experimentally, this roaming signal would overlap with the signal associated with the HCO product formation. As such, the amplitude observed in the DAS in this region between 8.5 and 9 eV would depend on the relative populations and cross sections of the two contributions, as the roaming component would provide a positive amplitude and the HCO component a negative amplitude.

The combined experimental and theoretical results lead to the following picture of the dynamics. Absorption of a UV photon leads to the population of the S_1_ excited state. Here, the C–C bond distance increases en route to a conical intersection, where there is efficient population transfer via a conical intersection to the *S*_0_ ground state,^10,16−18^ the extremely fast time scale rules out any intersystem crossing into the triplet state. Once in the ground state, a subset of the initially excited molecules continues along the dissociation path, leading to the rapid (50 fs) formation of the CH_3_ and HCO fragments. The remaining quasi-bound population is associated with roaming dynamics of the CH_3_ and HCO fragments, which explore a range of structures on the electronic ground state that potentially include those associated with the various ground-state minima. This roaming intermediate decays with a time constant of 190 ps, and we observe the concomitant formation of additional free HCO fragments on this time scale. We note that other roaming channels, involving a roaming hydrogen atom, could contribute to the signal and that the formation of other products, such as CH_4_+CO formed via reaction with the roaming HCO, or reformation of a vibrationally hot ground-state molecule, could also contribute to the effective lifetime measured. Detection of the CH_4_ + CO fragments is in principle also possible in the experiment due to the high probe energy used; however, the ionization potentials of these species mean they strongly overlap with the background associated with the ionization of ground-state CH_3_CHO. As we wish to stay in the regime where single-photon excitation is dominant, we are limited to a relatively low excited-state population and yield of products; this means we cannot extract reliable signals for these fragments.

Recent picosecond resolution action spectroscopy measurements of the products obtained following UV excitation suggest a much more complex set of electronic state transitions.^14,19^ The experiments were performed at a similar, albeit slightly lower pump energy (267 nm compared with 262 nm), which may explain the discrepancy. These action spectroscopy measurements suggested that the S_1_ lifetime was on the order of 100s ps, with complex excited-state dynamics over multiple electronic states required to explain the observation. We note that the evidence for the mechanisms is indirect, while our direct measurements capture the initial electronic state dynamics and ensure that processes exclusively associated with single-photon excitation are observed. The increased time resolution also shows that the S_1_ lifetime at our pump wavelength is less than 100 fs and leads to a relatively straightforward explanation of the dynamics.

## 3 Conclusions

The sensitivity of our XUV photoelectron spectroscopy probe allowed us to monitor the key geometric and electronic structure changes associated with roaming dynamics. We directly observed the formation of the roaming intermediate on an ultrafast (<100 fs) time scale. Detailed analysis of the resulting photoelectron spectrum and comparison to quantum chemical calculations allow us to show that roaming occurs on the electronic ground state and that the eventual breakdown of the roaming intermediate occurs on a 190 ps time scale. These experiments employing global probes with ultrafast time resolution offer the possibility for detailed and direct insight into molecular roaming pathways and for quantifying the importance and prevalence of roaming processes in photochemistry.

## Supporting Information Available

Experimental details and description of data analysis procedures and computational details and supporting calculations (PDF)
