# Supplementary material for: Direct Observation of a Roaming Intermediate and Its Dynamics
Source: J Am Chem Soc. 2024 Apr 29;146(18):12595–600. doi: 10.1021/jacs.4c01543 (PMC11082896; doi:10.1021/jacs.4c01543)
Supplement: Supplementary file 1 — ja4c01543_si_001.pdf [file ja4c01543_si_001.pdf]

Supplementary Information: Direct observation of a roaming intermediate and its dynamics

Grite L. Abma,[1] Michael A. Parkes,[2] Weronika O. Razmus,[3]
Yu Zhang,[4] Adam S. Wyatt,[4] Emma Springate,[4] Richard T. Chapman,[4]
Daniel A. Horke,[1*], Russell S. Minns[3*]

[1]Radboud University, Institute for Molecules and Materials,
Heijendaalseweg 135, 6525 AJ Nijmegen, The Netherlands
[2]Department of Chemistry, University College London, 20 Gordon Street, London
[3]School of Chemistry, University of Southampton,
Highfield, Southampton SO17 1BJ, UK
[4]Central Laser Facility, STFC Rutherford Appleton Laboratory,
Didcot, Oxfordshire OX11 0QX, UK

*To whom correspondence should be addressed;
DAH: d.horke@science.ru.nl, RSM: r.s.minns@soton.ac.uk.

Experimental Details

A detailed experimental setup has been published before, and only a brief overview is given here.[1] 1.5 bar of 2% acetaldehyde in helium (BOC speciality gases) is expanded into a molecular beam using a piezovalve operating at 1 kHz repetition rate. The produced molecular beam is skimmed once before entering the interaction chamber, where it is intersected by the pump and probe laser pulses. Produced photoelectrons are detected using an electron time-of-flight spectrometer (Kaesdorf ETF11).

Pump and probe laser pulses are derived from an amplified Ti:Sapphire laser system, operating at 1 kHz repetition rate (Red Dragon, KM Labs). The dispersion can be independently controlled in both beams using individual grating compressors. The pump beam at 262 nm is generated via subsequent second and third harmonic generation in beta-barium borate (BBO) crystals, which typically yielded 60 fs duration pulses at 10 μJ pulse energy. XUV probe pulses were generated via high-harmonic generation in an Argon gas-jet, driven by the second harmonic of the fundamental. The seventh harmonic of the drive laser (22.3 eV) is subsequently selected using a time-preserving monochromator[2], yielding 30 fs duration pulses with a typical flux of 10^{10} ph/s and a bandwidth of 130 meV (as detailed below). Pump and probe laser pulses are independently focused and then overlapped at a shallow angle (3 degrees) at the interaction point of the electron spectrometer. The pulse durations and crossing angle are all nominal, with the durations referring to approximate values at the point of generation. A more accurate measure of the combined contributions of the actual pulse duration at the interaction point are obtained from the fit through the instrument response function.

The data was collected in 3141 experimental cycles with a dwell time of 1 s per delay point in the cycle. Each experimental cycle consisted of 57 pump-probe delays. Each delay point is therefore the result of over 52 minutes of data collection corresponding to over 3 million laser

shots with a total collection time for the full data set of approximately 50 hours.

XUV bandwidth

The bandwidth of the XUV probe pulses was evaluated from the photoelectron spectrum of atomic Xenon, shown in Figure 1. The observed lines (corresponding to the $5p_{3/2}$ and $5p_{1/2}$ final states) were fitted with a Gaussian function, yielding bandwidth (FWHM) of 135 meV ($5p_{3/2}$) and 128 meV ($5p_{1/2}$).

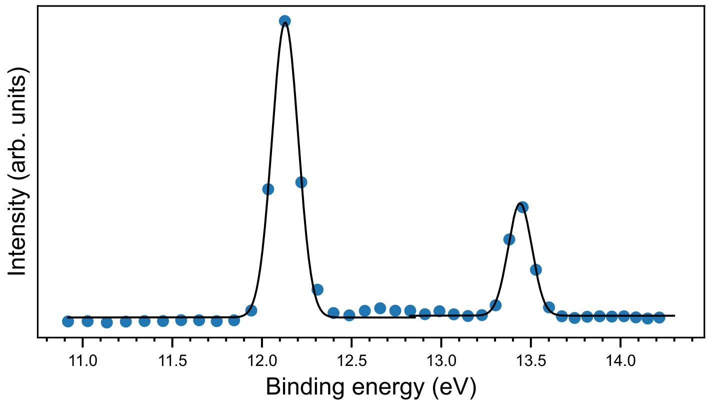

Figure 1: Photoelectron spectrum of atomic Xenon, with Gaussian fits to determine the harmonic bandwidth.

Photoelectron energy calibration

The energy of detected photoelectrons is calibrated by comparison with a published single-photon photoelectron spectrum for acetaldehyde[3]. A direct comparison between our data and previously published data is shown in Figure 2.

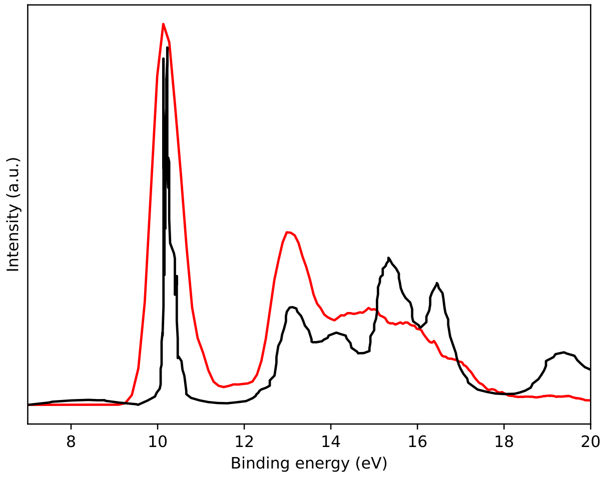

Figure 2: Overlay of the photoelectron spectrum measured using femtosecond XUV pulses (red trace) on an experimental He(II) photoelectron spectrum.[3] (black trace)

Data Analysis

Raw photoelectron spectra

The raw (non background subtracted) photoelectron spectra are shown in Figure 3 (on a logarithmic intensity scale) for three selected pump-probe delays. This clearly shows the relatively small excited-state signal compared to the ground state non-pumped signal, which arises from a combination of low excitation fraction and potentially lower ionisation cross-section of the excited state. For binding energies above ~ 9 eV any excited state (or product) signal is hidden within the large ground-state feature.

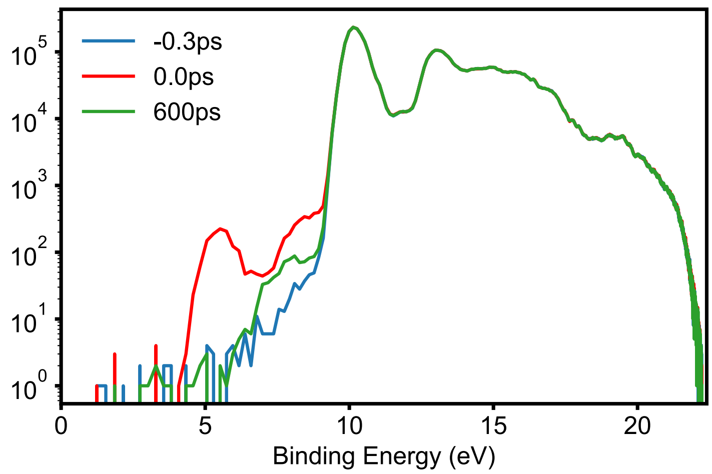

Figure 3: Photoelectron spectra of acetaldehyde for selected pump-probe delays.

Energy-integrated time-resolved plots

Figure 2 in the main paper shows the photoelectron intensity as a function of pump-probe delays integrated over 2 different photoelectron energy ranges. Statistical error bars for these data were evaluated by splitting the total collected dataset of 3141 experimental cycles into batches of 20 cyles, and evaluating the standard error on the collected photoelectron spectra. This error was then propagated to yield the shown error bars for energy-integrated traces. In figure 4 the same

data can be seen without normalisation.

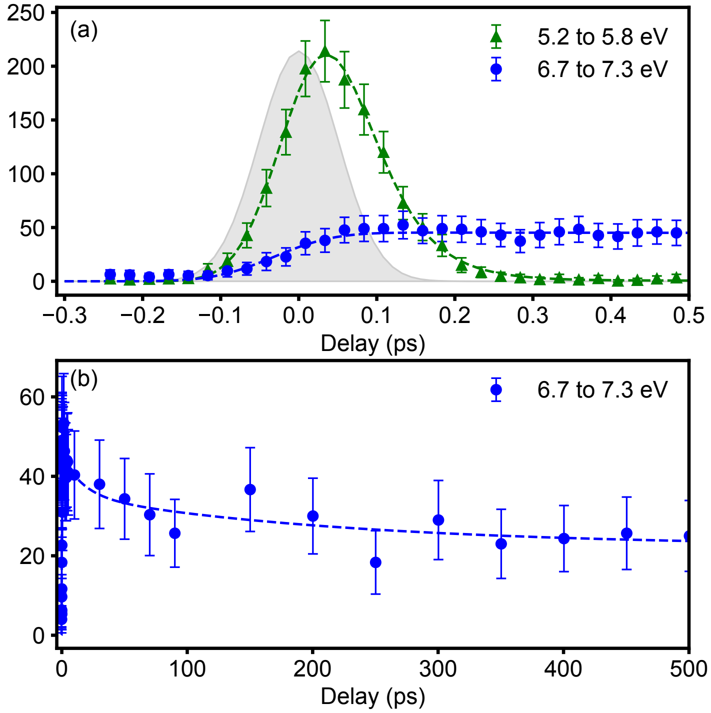

Figure 4: Time evolution of the observed photoelectron signal in selected energy regions on short (a) and long (b) timescales. Grey shading in (a) corresponds to the instrument response function. The data is equivalent to that presented in figure 2 of the main paper but without normalisation of the two signals.

Decay associated spectra

To deconvolve the various contributions to the spectrum we performed a 2D global least-squares fit to the data. The procedure simultaneously fits all photoelectron kinetic energies, ϵ_k, and time delays, t, to a function of the form

$$S(\epsilon_k, t) = g(t) \circledast \sum_{i=1}^{n} D_i(\epsilon_k) e^{-t/\tau_i}. \tag{1}$$

The fitting procedure assumes that the various configurations of the molecule produce a spectrum that is discrete and does not change in time. Within this assumption the fit then finds

the shape of the spectrum associated with each component and how their relative contributions to the overall spectrum changes over the course of the measurement. In equation 1, $D_i(\epsilon_k)$ represents the so called decay associated spectra (DAS) that provide the energy dependent amplitudes for each of the contributing components to the overall spectrum. These are convolved with a Gaussian, $g(t)$, that represents the instrument response function and multiplied by an exponential decay, $e^{-t/\tau}$ that describes how the intensity of the DAS changes with time. The fit is performed with the minimum number of terms in the sum required to obtain a good fit such that no systematic differences are observed in plots of the residual error. Here we required three separate time constants to accurately represent the experimental data. There are a total of 27 energy bins across the fitted region for all 57 pump-probe delays. The DAS fitting furthermore yielded an instrument response function (corresponding to the pump-probe cross-correlation) of 124 fs (FWHM).

The reconstructed 2D time-resolved photoelectron spectrum is shown in Figure 6, along with three spectra at selected time delays. For comparison, the experimental spectra at those time delays are shown as dashed lines. A very good match between the DAS fitting routine and experimental data is achieved, confirmed by the residual surfaces shown in Figure 7 and Figure 8.

To calculate confidence intervals for the parameters extracted from the DAS fitting we used a bootstrapping approach. 1000 test datasets were generated by adding random Gaussian-distributed noise to every datapoint, with the width of the Gaussian given by the average fractional residual. The latter is obtained by dividing the sum of the (absolute) residuals by the sum of the data, and was $9.7\ \%$. The DAS fitting routine is then run independently on those 1000 datasets, providing statistics and confidence intervals for the obtained fitting parameters. These are given in Table 1, with quoted errors corresponding to 2σ standard errors.

Table 1: parameters obtained from the DAS fitting and the errors in the fit obtained via a bootstrap analysis. The parameters define the three lifetimes of the DAS, the absolute position of time zero relative to the nominal position used during the experiment and the standard deviation, σ, of the Gaussian function that defines our instrument response.

Time Zero / fs	σ /fs	lifetime 1 /ps	lifetime 2 /fs	lifetime 3 /ps
-56.2 $\pm$ 0.2	52.5 $\pm$ 0.1	186 $\pm$ 6	47.9 $\pm$ 0.2	$>$2452

The fit shows that for the time zero, the instrument response function and the shortest lifetime no additional errors are added because of the fitting algorithm, the error in the fit is smaller than we expect the experimental error to be. For the lifetime of the roaming intermediate (time constant 1) we see that there is an uncertainty in the lifetime of several picoseconds. The nanosecond lifetime obtained is essentially infinite on the timescale of the measurement and defines a constant amplitude observed in the spectrum.

The errors on the amplitudes are very small as shown in Figure 5. In Figure 5 the shaded area around the solid lines represents the three sigma error obtained from the bootstrap analysis of the fit. We emphasise here that these only represent the error in the fit.

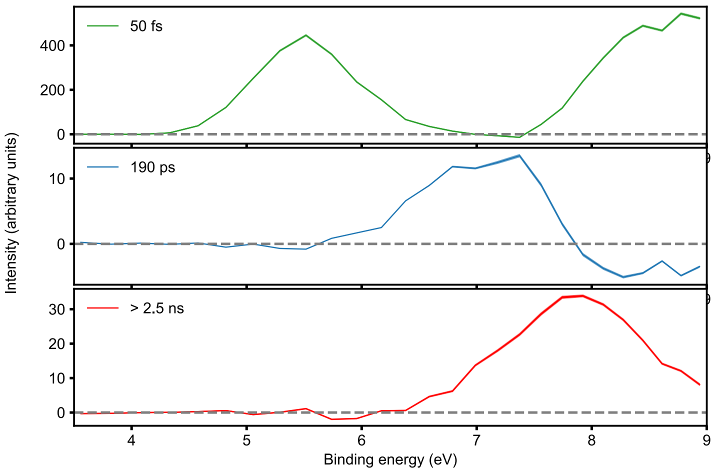

Figure 5: DAS with three sigma error from the fit represented by the line thickness.

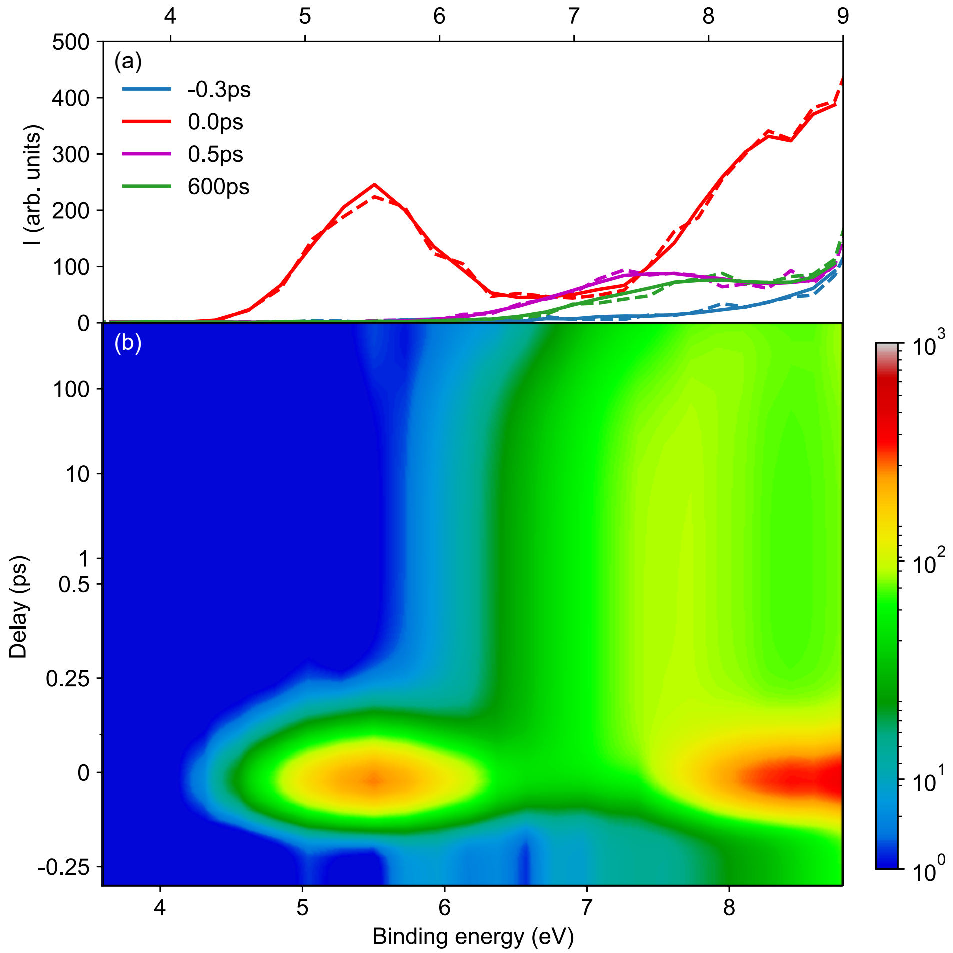

Figure 6: Results of the DAS fitting. (a) Extracted photoelectron spectra at four representative time delays extracted from the DAS fit (solid lines), and corresponding experimental spectra (dashed lines) for comparison. (b) The full 2D surface reconstructed by the DAS fit plotted on a mixed linear and logarithmic scale in time.

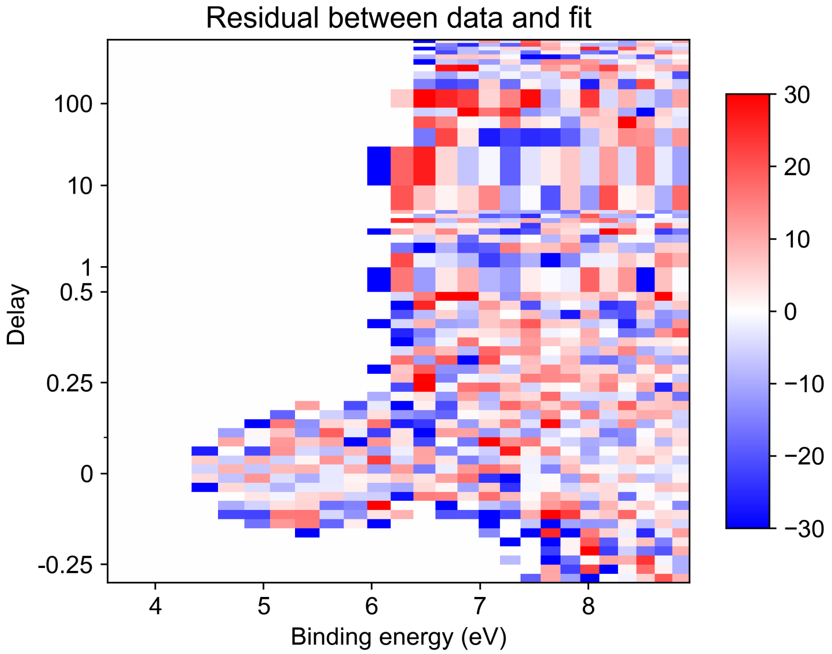

Figure 7: Residual surface comparing the DAS reconstructed 2D surfaces to the experimental data, difference given in percentage. Regions where the signal intensity is less than 3% of the maximum excited state signal are masked to zero.

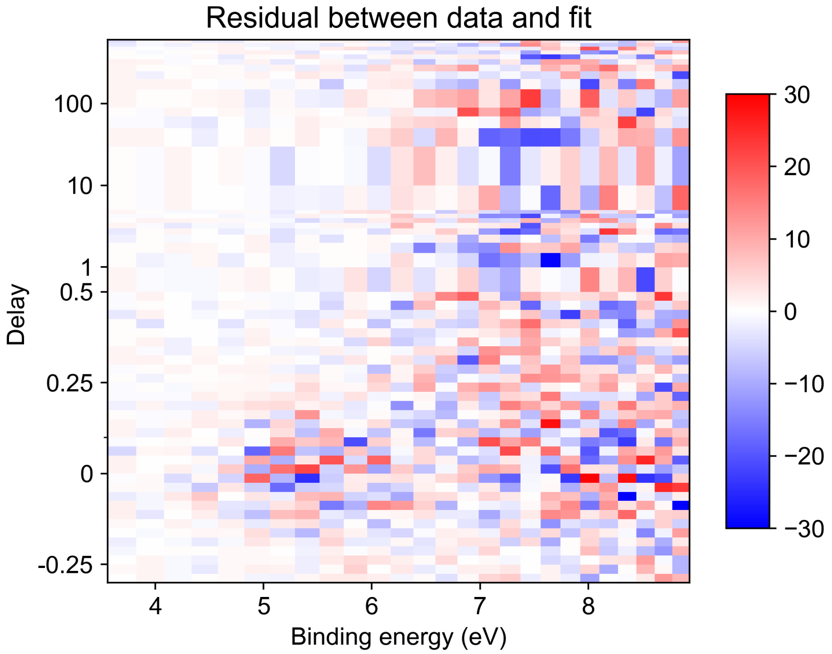

Figure 8: Residual surface comparing the DAS reconstructed 2D surfaces to the experimental data.

Computational

The vibrational frequencies and ground state structure of acetaldehyde were found by optimisation using the complete active space (CAS) self-consistent field method as implemented in the MolPro package.[4] The 6-311G** basis was used and the active space had 14 electrons and 13 orbitals. The orbitals included in the active space are shown in Figure 9. The ground state structure is shown in Table 2, while vibrational frequencies and their symmetries are given in Table 3.

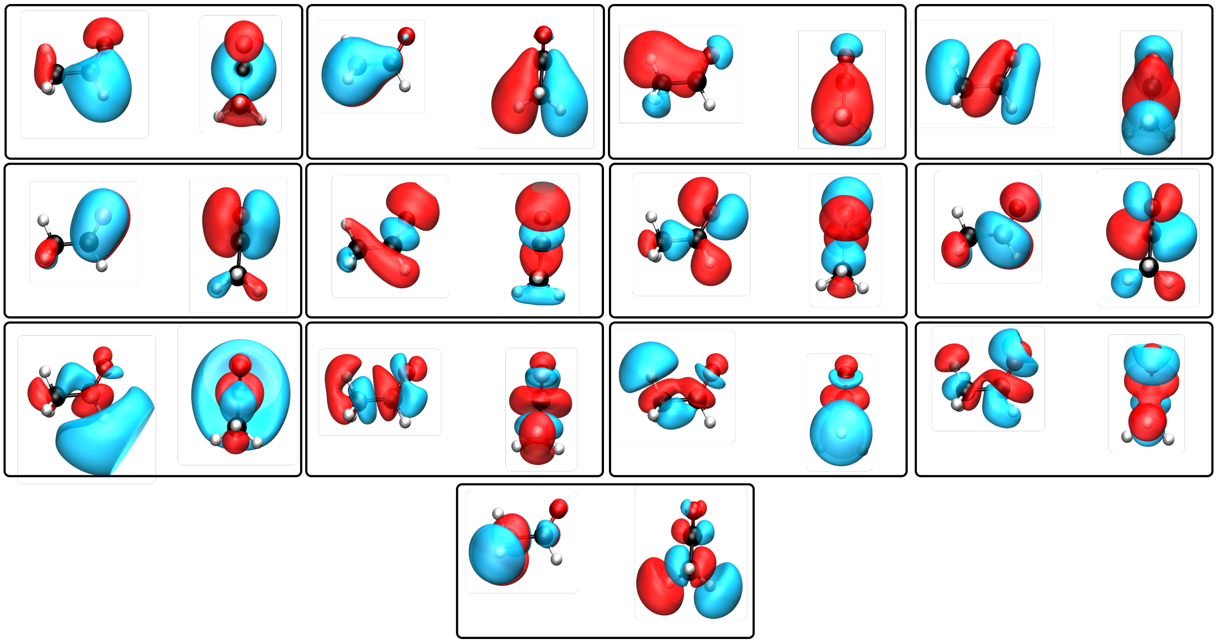

Figure 9: Molecular orbitals included in the CAS space.

Using the CAS ground state equilibrium structure state averaged CAS calculations were performed to examine the excited states of acetaldehyde. Initially 4 states were included in the state-averaged CAS calculation. But the energetic separation of the third and fourth states from the first two states was around 4 eV and examination of the surfaces showed that they did not come closer in energy. Therefore, we only included two states in the state-averaged CAS for

Atom	X /Å	Y /Å	Z /Å
O	-1.22396	0.366606	0
C	0.938853	-0.69637	0
C	-0.01404	0.468035	0
H	1.597243	-0.64969	0.891176
H	0.385637	-1.65162	0
H	1.597243	-0.64969	-0.89118
H	0.460675	1.459567	0

Table 2: Calculated ground state structure of acetaldehyde from a CAS calculation using the 6-311G** basis set.

further calculations. The (S_0) ground state has A' symmetry and the first excited state (S_1) has A" symmetry. Figure 10 shows a comparison of the calculated adiabatic energy between S_0 and S_1 and the experimental photoabsorption spectrum.[5] The calculated energy of S_1 relative to S_0 is 4.52 eV. As is clear the agreement between the calculated and measured transition energy is good.

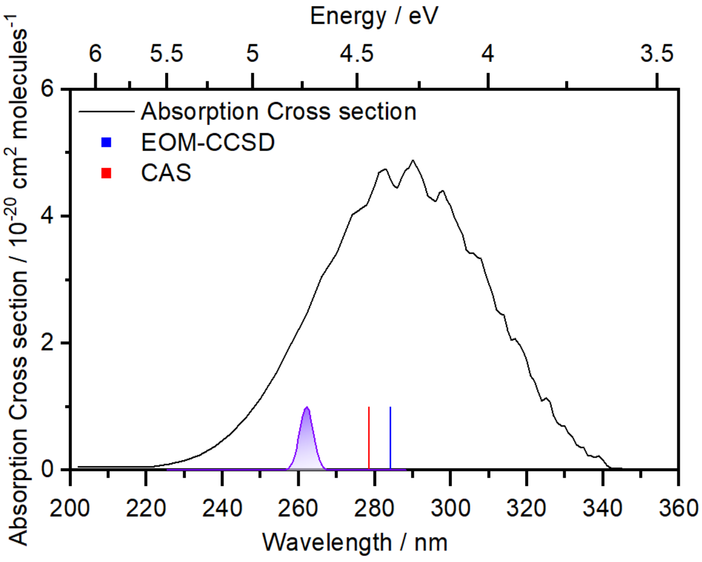

Figure 10: Comparison of experimental photoabsorption spectrum to calculated band positions using SA-CAS (14,13) and EOM-CCSD with the 6-311G** basis set. Experimental spectrum is taken from[5] the smaller gaussian represents the position of the pump beam relative to the absorption spectrum

Mode Number	Symmetry	Frequency / cm^-1
1	A”	155.36
2	A’	525.91
3	A”	820.37
4	A’	931.46
5	A’	1179.14
6	A”	1188.22
7	A’	1429.48
8	A’	1485.37
9	A”	1496.80
10	A’	1511.40
11	A’	1808.73
12	A’	2934.89
13	A”	2995.83
14	A’	3041.38
15	A’	3061.97

Table 3: Ground state frequencies of acetaldehyde from a CAS calculation using the 6-311G** basis set

Potential energy scans were performed to examine the S_0 and S_1 potential energy surfaces. Two types of scans were made. In the first a fixed scan was performed where all structural parameters were fixed apart from the C-C bond between the CH_3 and HCO moieties and the HCO angle. Relaxed scans were also performed along the C-C bond. The relaxed scans were performed minimising either the energy of the S_0 or the S_1 states. The results of these scans are shown in figure Figure 11 and in tables 4 and 5. From these scans key points on the potential energy surface were chosen for further calculations to be performed of ionization propensity. For these calculations the EOM-IP-CCSD method in Q-CHEM was used[6]. The calculated energy of S_1 using the EOM-CCSD method with the 6-311G** basis is shown in Figure 10 along with the value calculated using the CAS method. It can be seen that the EOM-CCSD result is in agreement with the CAS result, giving us confidence that (at least near the equilibrium structure) EOM-CCSD gives a good representation of the potential energy surface.

To test the reliability of the EOM-IP-CCSD method on our system, calculations were per-

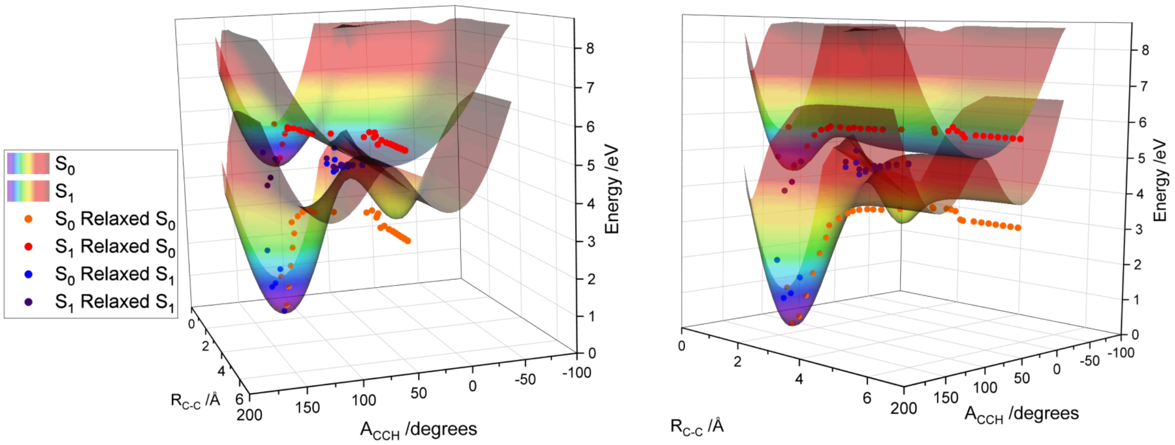

Figure 11: Potential energy surfaces of S_0 and S_1 of acetalaldhyde calculated using SA-CAS(14:13) and the 6-311G** basis set. Full surfaces are fixed scans, points represent the energies from relaxed scans. The orange and red dots are where the S_0 state is used as the basis of the optimisation, the blue and purple are where the S_1 state is the basis

formed on ionization of ground state acetaldehyde and the CO and CH_3 products. Figure 12 shows the results for acetaldehyde plotted against the experimental He(II) photoelectron spectrum of Bieri *et al*[3]. In Table 6 ionisation energies for the first three cation states of acetaldehyde, CH_3 and HCO are given with the experimental values. The experimental values for acetaldehyde are from Bieri *et al*[3], while those for CH_3 and HCO are from the NIST Chemistry Webbook[7]. As is clear there is good agreement between experiment and theory. There is a small shift of around 0.3 eV between the calculated and experimental position of the D_0 band of the spectrum while agreement is better for the higher-lying cation states.

For all the acetaldehyde geometries indicated on Figure 13 (S_0 and S_1 PES) the energies of the S_0, S_1, D_0 and D_1 states and the Dyson orbitals for the transitions were calculated using the EOM-IP-CCSD method with a 6-311G** basis. These orbitals were then used as input for calculation of the ionisation cross-sections using ezDyson[8;9]. The results of the calculations are then presented in Figure 14 where the differences between ionisation from the two electronic

R_{C-C} / Å	A_{CCH} / °	R_{C-O} /Å	A_{CCO} / °	S0 / eV	S1 / eV
1.3	115.02	1.11	127.00	1.08	5.66
1.5	115.44	1.11	124.75	0.09	4.56
1.7	114.43	1.11	123.60	0.33	4.73
1.9	113.31	1.10	122.99	0.96	5.16
2.1	112.27	1.10	122.86	1.63	5.50
2.3	111.50	1.10	122.99	2.21	5.66
2.5	111.27	1.10	122.43	2.68	5.71
2.7	115.30	1.10	119.12	3.07	5.78
2.9	110.96	1.10	124.16	3.27	5.77
3.1	108.01	1.10	127.20	3.43	5.75
3.3	109.63	1.10	125.71	3.52	5.79
3.5	109.42	1.09	126.14	3.58	5.78
3.7	107.43	1.09	128.26	3.62	5.79
3.9	108.02	1.09	127.74	3.64	5.79
4.1	106.09	1.09	129.71	3.65	5.80
4.3	90.59	1.09	145.23	3.66	5.80
4.5	56.20	1.09	180.39	3.66	5.80
4.7	64.00	1.10	169.07	3.67	5.71
4.9	55.33	1.09	180.80	3.66	5.76
5.1	58.67	1.08	181.28	3.68	5.89
5.3	62.09	1.09	186.41	3.67	5.80
5.3	55.12	1.09	180.15	3.42	5.71
5.5	61.21	1.09	191.76	3.44	5.64
5.7	55.94	1.09	181.49	3.43	5.71
5.9	55.25	1.09	180.34	3.43	5.71
6.1	55.20	1.09	180.29	3.43	5.71
6.3	55.37	1.09	179.51	3.43	5.71
6.5	55.34	1.09	179.55	3.43	5.71
6.7	55.31	1.09	179.60	3.43	5.71
6.9	55.26	1.09	179.66	3.43	5.71

Table 4: Geometries and energies for relaxed scan of the C-C bond length. Optimisation is performed on the S_0 surface

states are clear.

To further understand the early time dynamics we have performed quantum molecular dynamics simulations over the first 100 fs to confirm ultrafast IC is possible. The simulations were performed using the Direct Dynamics variational Multi-configurational Gaussian method

R_{C-C} / Å	A_{CCH} / °	R_{C-O} /Å	A_{CCO} / °	S0 / eV	S1 / eV
1.3	128.01	1.13	119.79	1.92	4.85
1.5	126.66	1.11	117.66	0.86	3.91
1.7	124.93	1.10	115.87	1.03	4.17
1.9	121.17	1.10	113.93	1.49	4.75
2.1	71.06	1.10	103.20	4.54	4.69
2.3	65.09	1.06	105.47	4.67	5.02
2.5	68.95	1.12	108.70	4.37	4.52
2.7	68.86	1.10	109.21	4.46	4.51
2.9	65.75	1.07	112.02	4.60	4.64
3.1	66.28	1.10	114.34	4.60	4.66
3.3	63.74	1.09	118.10	4.65	4.72
3.5	61.24	1.10	118.98	4.72	4.73
3.7	54.13	1.10	126.16	4.75	4.77

Table 5: Geometries and energies for relaxed scan of the C-C bond length. Optimisation is performed on the S_1 surface

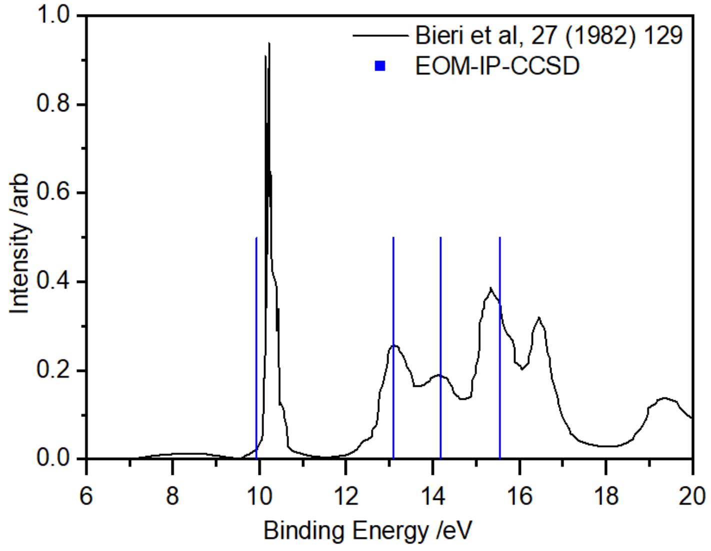

Figure 12: Comparison of experimental He(II) photoelectron spectrum to calculated binding energies using EOM-IP-CCSD.[3]

(DD-vMCG).[10;11] In this method the nuclear wave function is represented by a superposition of Gaussian wavepackets (GWP) that follow variationally coupled trajectories. The potential energy surface that the trajectories travel upon was calculated *on the fly*, the surface was calcu-

	EOM-IP-CCSD / eV	Expt / eV
Acetaldehyde D_0	9.93	10.22
Acetaldehyde D_1	13.09	13.05
Aceetaldehyde D_2	14.18	14.15
CH_3	9.55	9.84
HCO	8.97	8.12

Table 6: Comparison of experimental and calculated ionization energies

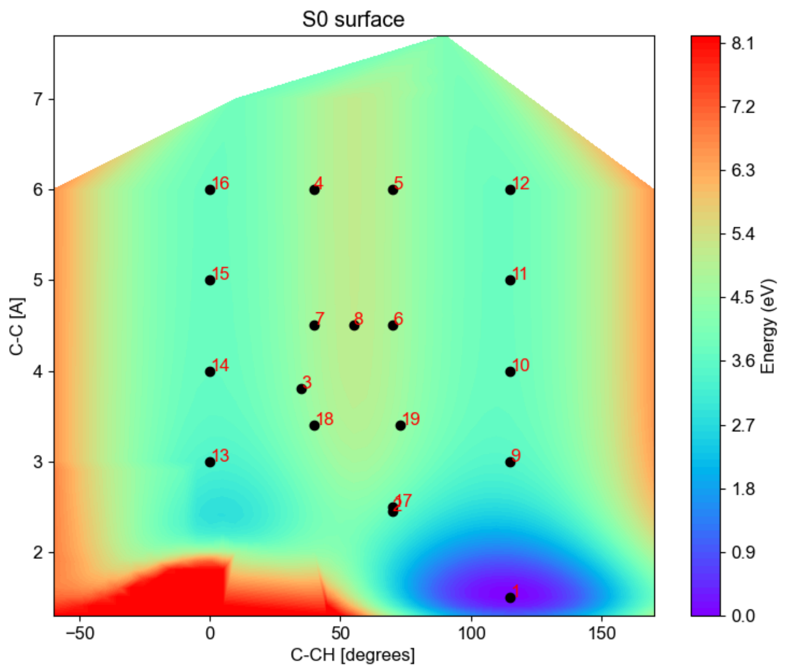

Figure 13: The calculated S0 surface with the points at which the dyson calculations were performed drawn in.

lated using our benckmarked two state SA-CAS(14:13) with a 6-311G** basis set in Molpro. The DD-vMCG calculations were performed using the Quantics software package.[12] A total of 30 GWPs were required to model the 100 fs timeframe covered.

The initial step in the simulation is to transfer population from the ground state onto the excited state. The projection samples the full ground state vibrational distribution and is therefore

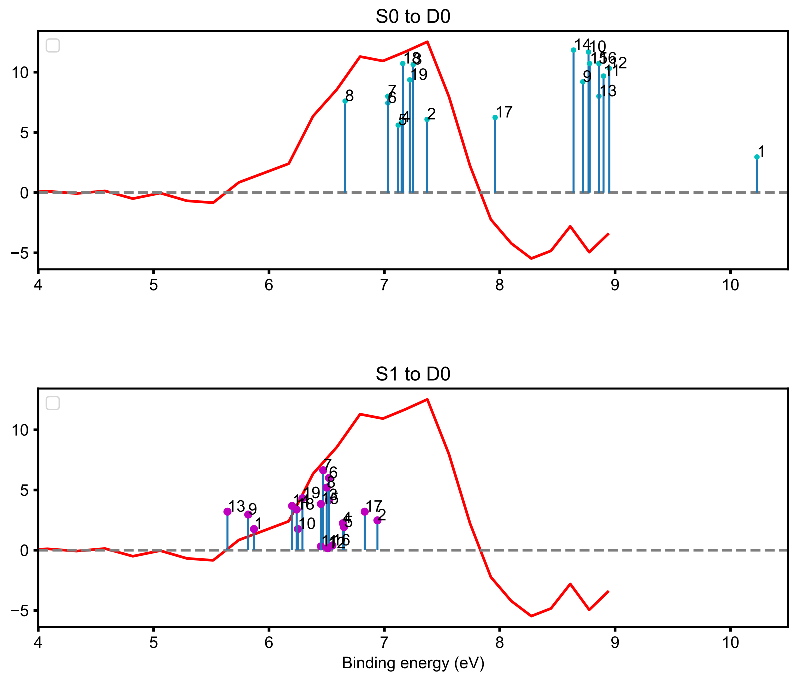

Figure 14: The energies of all calculated points on the S_0 and S_1 surface, with the number indicating to which point on the surface the energy corresponds. The length of the stick corresponds to the cross section of this transition.

representing excitation across the entire absorption band presented in Figure 10. This means the results provide data that is a weighted average of the dynamics possible at all excitation energies that populate S_1. The results, while useful in defining what processes are possible and on what timescales these occur, cannot be used to quantify the relative importance of a particular process at a given excitation energy. With this in mind, in Figure 15 we plot the populations of S_0 and S_1 over the 100 fs of the simulation. The simulations show that $\sim$10 % of the excited state population in S_1 undergoes ultrafast IC to S_0 within 20 fs. After this initial step there is little change in the relative populations of the two states for the remainder of the 100 fs sampled. While the trajectory calculations cannot confirm the relative importance of this channel at our excitation wavelength, they do confirm that upon excitation to S_1 ultrafast IC to S_0 is possible

on the few 10’s of femtoseconds timescales observed in the experiment.

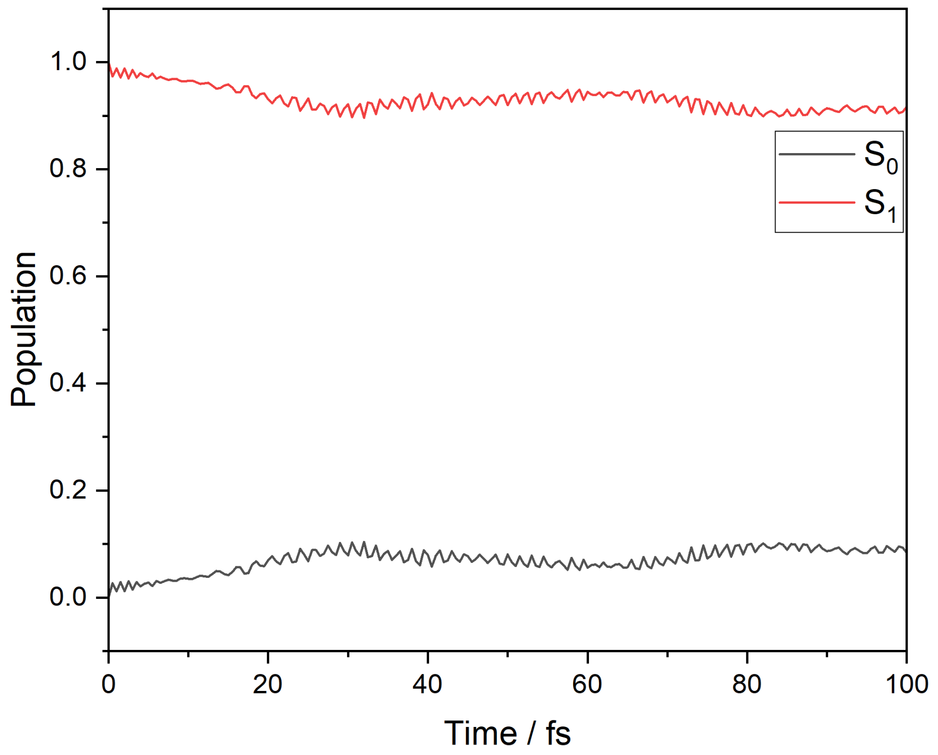

Figure 15: Populations of the S_0 and S_1 states of acetaldehyde as a function of time after initial excitation calculated using the DD-vMCG method.

References

[1] Smith, A. D.; Warne, E. M.; Bellshaw, D.; Horke, D. A.; Tudorovskya, M.; Springate, E.; Jones, A. J. H.; Cacho, C.; Chapman, R. T.; Kirrander, A.; Minns, R. S. Mapping the Complete Reaction Path of a Complex Photochemical Reaction. *Phys. Rev. Lett.* **2018**, *120*, 183003.

[2] Frassetto, F.; Cacho, C.; Froud, C. A.; Turcu, I. E.; Villoresi, P.; Bryan, W. A.; Springate, E.; Poletto, L. Single-grating monochromator for extreme-ultraviolet ultrashort pulses. *Opt. Express* **2011**, *19*, 19169–19181.

[3] Bieri, G.; Åsbrink, L.; von Niessen, W. 30.4-Nm He (II) Photoelectron Spectra of Organic Molecules. *J. Electron. Spectros. Relat. Phenomena* **1982**, *27*, 129–178.

[4] Werner, H.-J. et al. MOLPRO, version 2015.1, a package of ab initio programs. 2015.

[5] Keller-Rudek, H.; Moortgat, G. K.; Sander, R.; Sörensen, R. The MPI-Mainz UV/VIS Spectral Atlas of Gaseous Molecules of Atmospheric Interest. *Earth Syst. Sci. Data* **2013**, *5*, 365.

[6] Shao, Y. et al. Advances in Molecular Quantum Chemistry Contained in the Q-Chem 4 Program Package. *Mol. Phys.* **2015**, *113*, 184–215.

[7] Lias, S. G. In *NIST Chemistry WebBook, NIST Standard Reference Database Number 69*; Linstrom, P. J., Mallard, W. G., Eds.; National Institute of Standards and Technology: Gaithersburg , MD, 2022.

[8] Gozem, S.; Krylov, A. I. The *ezSpectra* Suite: An Easy-to-use Toolkit for Spectroscopy Modeling. *WIREs Comput Mol Sci* **2022**, *12*.

[9] Gozem, S.; Gunina, A. O.; Ichino, T.; Osborn, D. L.; Stanton, J. F.; Krylov, A. I. Photoelectron Wave Function in Photoionization: Plane Wave or Coulomb Wave? *J. Phys. Chem. Lett.* **2015**, *6*, 4532–4540.

[10] W, R. G.; Polyak, I.; Spinlove, K. E.; Worth, G. A.; Burghardt, I.; Lasorne, B. Quantum dynamics simulations using Gaussian wavepackets: the vMCG method. *International Reviews in Physical Chemistry* **2015**, *34*, 269–308.

[11] Christopoulou, G.; Freibert, A.; Worth, G. A. Improved algorithm for the direct dynamics variational multi-configurational Gaussian method. *The Journal of Chemical Physics* **2021**, *154*, 124127.

[12] Worth, G. Quantics: A general purpose package for Quantum molecular dynamics simulations. *Computer Physics Communications* **2020**, *248*, 107040.
